# New technique for the treatment of fresh bony mallet finger: A retrospective case series study

**DOI:** 10.3389/fsurg.2023.1127827

**Published:** 2023-03-29

**Authors:** Zhenshuang Yue, Yafeng Mo, Zhenfei Xiong, Yanghua Tang

**Affiliations:** ^1^ Department of Orthopedics, Hospital of Traditional Chinese Medicine of Xiaoshan District, Hangzhou, China; ^2^ Zhejiang Rehabilitation Medical Center, Hangzhou, China

**Keywords:** bony mallet finger, kirschner wire, tendon, fracture, phalanx

## Abstract

**Background:**

The bony mallet finger is a tear fracture of the extensor tendon, resulting in a flexion deformity of the finger, which affects both the function of the finger. The classical Ishiguro's method is associated with damage to the cartilage of the distal interphalangeal (DIP) joint and always lead to the joint stiffness. This paper explores a new technique to overcome the shortcomings of the classical Ishiguro's method and achieve better clinical efficacy.

**Methods:**

We examined 15 patients with bony mallet fingers, 9 males and 6 females, from February 2020 to June 2022, ranged from 23 to 58 years, including 1 case of index finger, 5 cases of middle finger, 3 cases of ring finger and 6 cases of little finger. The median course of the injury to surgery was 2 days (range, 1∼7 days). All had fresh closed injuries, according to the Wehbe and Schneider classification: 4 cases of type IA, 6 cases of type IB, 3 cases of type IIA and 2 cases of type IIB. All patients were treated surgically by the new technique. Post-operative follow-up was conducted to record the healing of the fracture, the pain of the affected finger and the function of joint movement.

**Results:**

The 15 cases were followed up after surgery. The median active range of motion was 65° (range, 55∼75°). The median extension deficit of DIP joint was 0° (range, 0∼11°). The median clinical healing time of the fracture was 6 weeks (range, 6∼10 weeks). None of the patients experienced significant pain. The patients were assessed according to the Crawford criteria at the final follow-up: 11 cases were assessed as excellent, 3 cases were assessed as good and 1 case was assessed as fair. No loss of fracture repositioning, loosening of internal fixation, skin necrosis or infection was observed.

**Conclusion:**

The application of the new technique for the treatment of bony mallet fingers has the advantages of good stability, fracture healing and functional recovery of the DIP joint, making it an ideal surgical procedure for the treatment of fresh bony mallet fingers.

## Introduction

Bony mallet finger is a common hand trauma, which is characterized by swelling and pain in the distal interphalangeal (DIP) joint of the affected finger. Without the follow-up treatment, it may lead to a flexion deformity, and even a swan-neck deformity of the finger. The treatment of the bony mallet finger currently consists of conservative and surgical treatment. Because of the high failure rate of conservative treatment, more and more surgeons are advocating early surgical intervention after injury. The most classic form of the procedure is the Ishiguro's method, which has been reported to be effective, but there are still some shortcomings, such as stiffness of joints and cartilage damage (1, 2). So we modified the design of the method. From February 2018 to June 2019, this study successfully repaired bony mallet finger using the new technique in 15 patients. After the patient's fracture had healed and then Kirschner wires (*K*-wires) were removed, the DIP joint function was noted on follow-up.

## Methods

### Patients

15 patients (9 males and 6 females) were recruited for the study, whose ages ranged from 23 to 58 years, with a median age of 39 years, including 1 case presented on the index finger, 5 on the middle finger, 3 on the ring finger and 6 on the little finger. The course of injury to surgery ranged from 1 to 7 days, with a median course of 2 days. All were fresh closed injuries, according to the Wehbe-Schneider classification (Table 1) (3): 4 cases of type IA, 6 cases of type IB, 3 cases of type IIA and 2 cases of type IIB.

**Table 1:** Demographics of 15 patients.

Patient No.	Age	Gender	Hand	Finger	Mechanism of injury	Wehbe and Schneider's calssification	Injury to surgery (days)	Follow-up (weeks)	VAS	Extension Deficit (*n*°)	AROM (*n*°)	Crawford Criteria	Complication	Healing time of fracture (weeks)
1	23	Male	R	2	Basketball	IA	2	14	0	0	70	Excellent	No	6
2	25	Male	L	5	Basketball	IB	5	12	0	0	62	Excellent	No	6
3	45	Female	R	3	Traffic accident	IIA	2	18	0	0	65	Excellent	No	8
4	34	Male	L	4	Basketball	IA	5	22	0	0	60	Excellent	No	6
5	37	Female	L	5	Work	IA	1	16	0	0	75	Excellent	No	6
6	45	Male	R	4	Fight	IIA	2	16	0	0	68	Excellent	No	6
7	50	Female	R	3	Work	IB	2	24	0	11	60	Fair	No	8
8	37	Male	R	5	Fall	IB	3	16	0	6	65	Good	No	6
9	25	Male	L	3	Basketball	IA	3	18	0	10	55	Good	No	6
10	39	Female	R	5	Traffic accident	IIB	7	24	0	0	68	Excellent	No	8
11	42	Male	L	3	Work	IB	6	28	0	0	65	Excellent	No	8
12	43	Female	R	5	Work	IB	3	30	0	8	65	Good	No	6
13	32	Male	L	5	Basketball	IIB	1	26	0	0	70	Excellent	No	8
14	55	Male	R	3	Traffic accident	IB	2	28	0	0	60	Excellent	No	10
15	58	Female	L	4	Fall	IIA	1	16	0	0	70	Excellent	No	8

### Treatment method

#### The operation of surgery

**Figure 1:**
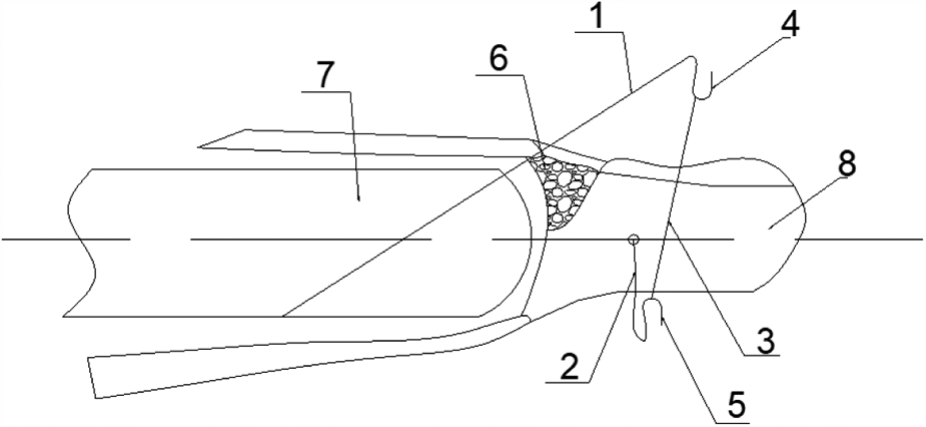
A schematic diagram of the new technique (1. Blocking wire; 2. *K*-wire; 3. Rubber band; 4. Hook; 5. Hook; 6. Avulsion fracture; 7. Middle phalanx; 8. Distal phalanx).

After anaesthesia, disinfection, draping, the DIP joint of the affected finger was hyperextended to reposition the avulsed bone under C-arm fluoroscopy, with the aid of a 0.8 mm diameter *K*-wire if necessary. To determine keeping the maximum flexion of the DIP joint after repositioning, a *K*-wire with a diameter of 1.0 mm or 1.2 mm was used to drill into the middle phalanx from the dorsal side of the distal fracture block (without penetrating into the fracture block) at an angle of 35°∼45° with the axis of the middle phalanx. It was appropriate to drill the wire just through the middle phalanx cortex. In order to avoid damage to the articular cartilage of the middle phalanx and the skin of the finger caused by repeated drilling, the drilling point of the *K*-wire was determined by fluoroscopic positioning of a syringe needle before insertion of the *K*-wire. The end of the *K*-wire was cut off and folded into a curved hook. The end of the *K*-wire should not exceed 1/4 of the distal finger. The *K*-wire was used as a “blocking wire”. The distal phalanx was pulled distally and the DIP joint was straightened to reposition the torn bone block. After confirming that the fracture was well repositioned and the position of the blocking wire was appropriate, another 0.8 mm *K*-wire was penetrated perpendicularly from the side of the distal phalanx to the contralateral side. Bend both ends of the *K*-wire at 3 mm from the skin, and fold the two ends of the wire respectively into a curved hook for use. Connect the bend hook of blocking wire and the transverse *K*-wire with the rubber band (made by polyisoprene), adjust the tension of the rubber band, and make the DIP joint fixed at the dorsiflexion position of 5°∼10°. The surgery was completed after fluoroscopy again, which confirmed that the fracture block was in good position. A schematic diagram of the new technique is shown in the Figure 1.

### Postoperative management

After operation, the patient was instructed to disinfect the wire-hole with alcohol to prevent infection. X-rays were reviewed to observe fracture repositioning and healing every 2 weeks. The patients were instructed to perform DIP joint active functional exercise in a small range 2 weeks after operation. The elasticity of the rubber band was adjusted every 2 weeks after surgery to fix the DIP joint in the straight position. In the 6th∼10th week after surgery, the decision to remove the *K*-wire was made according to the stability of fixation and fracture healing. Then active and passive functional exercises of the DIP joint were performed under the guidance of the physician.

### Efficacy evaluation index

Follow-up examinations were arranged every 2 weeks to evaluate complications including malunion, skin necrosis, and infection based on physical examination and imaging. The main indexes collected were the pain of the finger, appearance of the finger and the function of the joint. The final follow-up evaluation indexes were assessed according to the Crawford criteria (Table 1) (4).

## Results

The 15 cases were followed up for 12 to 30 weeks after surgery, with a median time of 18 weeks. The median active range of motion (AROM) was 65° (range, 55∼75°). The median extension deficit of the DIP joint was 0° (range, 0∼11°). The median clinical healing time of the fracture was 6 weeks (range, 6∼10 weeks). None of the patients experienced significant pain. The patients were assessed according to the Crawford criteria at the final follow-up: 11 cases were assessed as excellent, 3 cases were assessed as good and 1 case was assessed as fair. No loss of fracture repositioning, loosening of internal fixation, skin necrosis or infection was observed. The results of the new technique can be seen in the Table 1.

### Typical case

**Figure 2:**
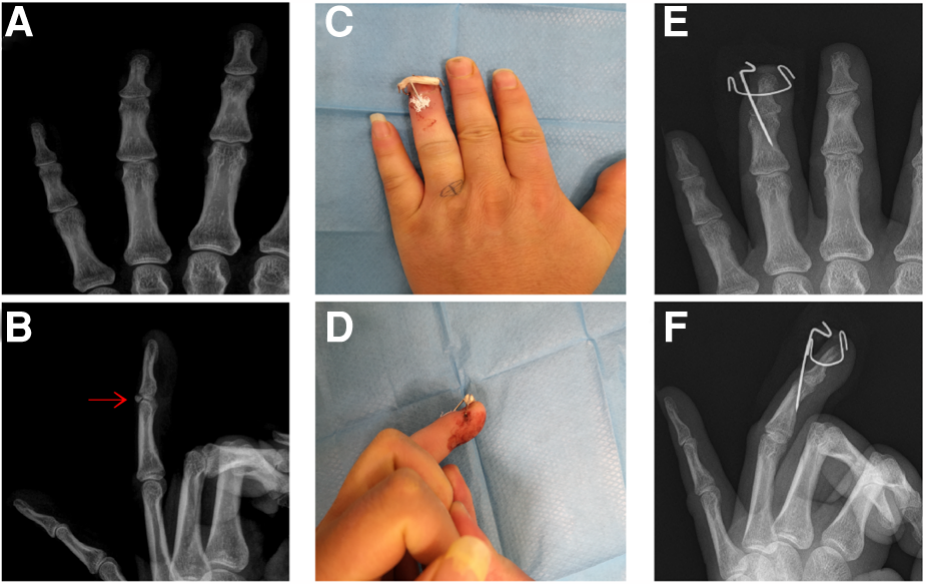
Preoperative and postoperative data of patient No.4. (**A,B**). Preoperative X-ray; the avulsion fracture (red arrow). (**C,D**). Postoperative X-ray. (**E,F**). Postoperative appearance.

**Figure 3:**
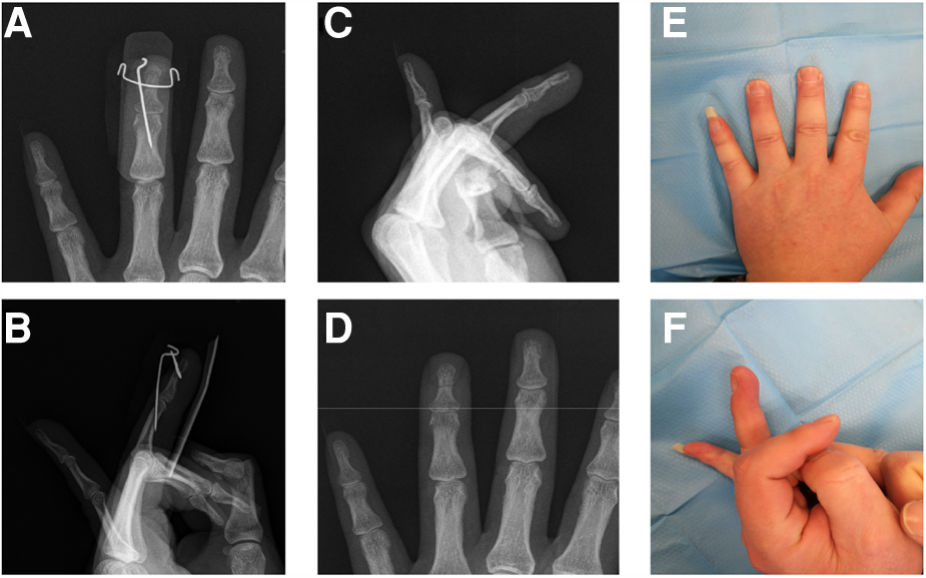
Follow-up of patient No. 4. (**A,B**). X-ray at 6 weeks postoperatively. (**C,D**). X-ray at 22 weeks postoperatively. (**E,F**). Finger appearance at 22 weeks.

A 34-year-old male came to our hospital for 4 days due to limited extension of his left ring finger after playing basketball. The affected finger was temporarily fixed by splint. X-ray examination was performed to confirm the diagnosis of bony mallet finger (Figures 2A,B). The next day, the patient was treated with the new technique under fluoroscopy (Figures 2C,D). Postoperative X-ray examination was performed (Figures 2E,F). After discharge, the patient was informed to review the X-ray at the outpatient clinic every 2 weeks. X-ray examination 6 weeks later showed that the fracture had healed and the *K*-wires were removed (Figures 3A,B). Then the patient was informed to review every 4 weeks. The recovery of finger function was evaluated at the last follow-up (Table 1 Patient No. 4, Figures 3C–F).

### Failure case

**Figure 4:**
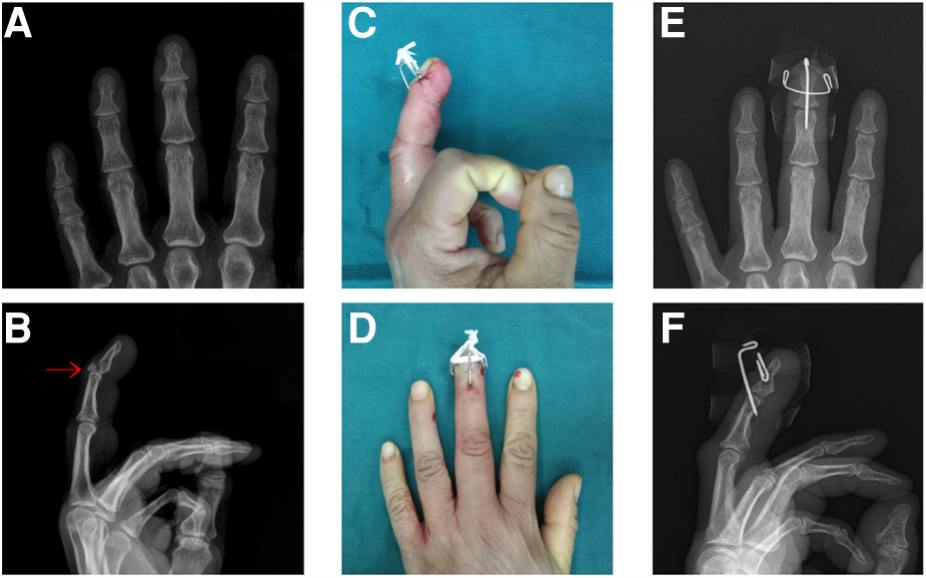
Preoperative and postoperative data of patient No.7. (**A,B**). Preoperative X-ray. (**C,D**). Postoperative X-ray. (**E,F**). Postoperative appearance.

**Figure 5:**
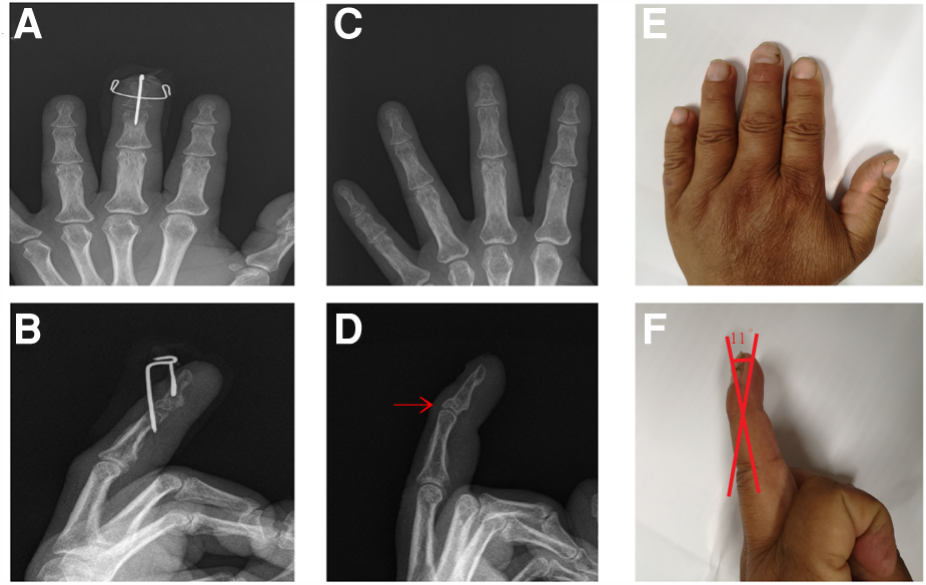
Follow-up of patient No.7. (**A,B**). X-ray at 8 weeks postoperatively. (**C,D**). X-ray at 24 weeks postoperatively;malunion (red arrow). (**E,F**). Finger appearance at 24 weeks; extension deficit (red angle).

A 50-year-old female came to our hospital for 1 day due to limited extension of her right middle finger after agricultural work. The affected finger was temporarily fixed by splint. X-ray examination was performed to confirm the diagnosis of bony mallet finger (Figures 4A,B). The next day, the patient was treated with the new technique under fluoroscopy (Figures 4C,D). Postoperative X-ray examination was performed (Figures 4E,F). After discharge, the patient was informed to review the X-ray at the outpatient clinic every 2 weeks. The patient was absent for 6 weeks due to family reasons after 1 review. When she returned to the hospital, X-ray examination showed that the fracture had healed, but the rubber band was relaxed (Figures 5A,B). And then the *K*-wires were removed. Then the patient was informed to review every 4 weeks. The recovery of finger function was evaluated at the last follow-up (Table 1 Patient No. 7). The distal phalanx of the patient had a malunion and the finger of the patient had an extension deficit (Figures 5C–F).

## Discussion

If not effectively treated in a timely manner, bony mallet finger can eventually lead to instability of the DIP joint, osteoarthritis and degenerative arthritis, or even a “swan-neck” deformity, which can seriously affect the function and appearance of the finger and cause great inconvenience to the patient's life and work (5, 6). The early treatment of the bony mallet finger is of great importance in promoting functional rehabilitation and reducing complications, which is also in line with the demands of modern medical technology.

The principles of treatment are aimed at reestablishing the stability of the DIP joint, repairing the fracture, restoring the function of the extensor tendon to balance the forces of flexion and extension (7, 8). The treatment of the bony mallet finger should restore the anatomy of the DIP joint by repositioning and fixing the avulsion fracture firstly, and secondly maintain the stability of the DIP joint by resisting the strong finger flexion forces (9, 10). The “Ishiguro method” requires fixation of the DIP joint with a wire, which can cause damage to the articular surface and complications such as nail deformity and joint stiffness (11, 12). We have modified the “Ishiguro method” and designed it. The procedure consists of 2 *K*-wires and 1 rubber band, in which the wires don't need to be fixed the DIP joint to avoid damage to the articular surface and to allow early active and passive functional exercise of the finger.

The advantages of this new technique include: (i) No damage to the epiphyseal plate and articular surface, which can avoid secondary damage such as articular cartilage degeneration and joint capsule contracture; (ii) Antagonism of the finger flexor tendon force can balance the flexion and extension forces of the distal phalanx to facilitate the healing of the fracture; (iii) Early functional exercise of the DIP joint under the protection of the elastic external fixation to avoid joint stiffness.

The following should be noted when using the new technique for the treatment of fresh bony mallet finger: (i) Before placing the blocking wire, the point of entry should be determined by syringe needle to avoid repeated damage to the articular cartilage surface of the middle phalanx and the skin of the finger. (ii) The entry angle of the blocking wire should be controlled at 35°∼45°, a small angle can cause skin compression and nail bed damage easily, and a large angle will weaken the blocking and fixation effect of the avulsed fracture conversely. (iii) The blocking wire should be inserted just above the volar cortex, a long wire can damage the flexor tendon and affect its movement, which is not conducive to finger rehabilitation. (iv) An appropriate amount of cotton balls can be placed at the point of contact between the blocking wire and the dorsal skin of the finger to avoid skin compression by the blocking wire. (v) Patients must be required for strict needle tract care to prevent infection. (vi) Regular outpatient review to observe fracture repositioning and healing, and adjustment should be made in the event of loss of fracture repositioning and loosening of the wires or rubber band.

## Limitations

The limitations of this technique are: (i) It is not suitable for those with a disease duration of more than 3 week, as reposition is difficult. (ii) The blocking wire is a point blocking fixation, which is only suitable for single avulsion fractures (Wehbe-Schneider types Ia, Ib, IIa and IIb) with relatively large and intact bones, but cannot be effectively fixed for comminuted fractures; (iii) The patient must have good compliance, otherwise it is prone to tract infection, loosening of the blocking wire and loss of bone repositioning.

The sample size of this study is small and the follow-up period is short. We will also include a control group in subsequent studies to further clarify the advantages of the new technique.

## Data Availability

The original contributions presented in the study are included in the article/Supplementary Material, further inquiries can be directed to the corresponding author.
